# miRspongeR 2.0: an enhanced R package for exploring miRNA sponge regulation

**DOI:** 10.1093/bioadv/vbac063

**Published:** 2022-09-02

**Authors:** Junpeng Zhang, Lin Liu, Wu Zhang, Xiaomei Li, Chunwen Zhao, Sijing Li, Jiuyong Li, Thuc Duy Le

**Affiliations:** Department of Information and Electronic Engineering, School of Engineering, Dali University, Dali 671003, China; UniSA STEM, University of South Australia, Mawson Lakes, SA 5095, Australia; Department of Molecular Biology, School of Agriculture and Biological Sciences, Dali University, Dali 671003, China; UniSA STEM, University of South Australia, Mawson Lakes, SA 5095, Australia; Department of Information and Electronic Engineering, School of Engineering, Dali University, Dali 671003, China; Department of Information and Electronic Engineering, School of Engineering, Dali University, Dali 671003, China; UniSA STEM, University of South Australia, Mawson Lakes, SA 5095, Australia; UniSA STEM, University of South Australia, Mawson Lakes, SA 5095, Australia

## Abstract

**Summary:**

MicroRNA (miRNA) sponges influence the capability of miRNA-mediated gene silencing by competing for shared miRNA response elements and play significant roles in many physiological and pathological processes. It has been proved that computational or dry-lab approaches are useful to guide wet-lab experiments for uncovering miRNA sponge regulation. However, all of the existing tools only allow the analysis of miRNA sponge regulation regarding a group of samples, rather than the miRNA sponge regulation unique to individual samples. Furthermore, most existing tools do not allow parallel computing for the fast identification of miRNA sponge regulation. Here, we present an enhanced version of our R/Bioconductor package, miRspongeR 2.0. Compared with the original version introduced in 2019, this package extends the resolution of miRNA sponge regulation from the multi-sample level to the single-sample level. Moreover, it supports the identification of miRNA sponge networks using parallel computing, and the construction of sample–sample correlation networks. It also provides more computational methods to infer miRNA sponge regulation and expands the ground truth for validation. With these new features, we anticipate that miRspongeR 2.0 will further accelerate the research on miRNA sponges with higher resolution and more utilities.

**Availability and implementation:**

http://bioconductor.org/packages/miRspongeR/.

**Supplementary information:**

Supplementary data are available at *Bioinformatics Advances* online.

## 1. Introduction

By competing for shared microRNA (miRNA) response elements (MREs), diverse coding and non-coding RNA transcripts act as miRNA sponges, also called competing endogenous RNAs (ceRNAs) or miRNA decoys, to separate miRNAs from binding with messenger RNAs (mRNAs) (Salmena *et al.*, 2011). As the main type of regulators of miRNA activities, miRNA sponges play an important role in numerous biological processes, including the initiation and progression of cancers (Tay *et al.*, 2014).

For the majority of contemporary biomedical studies, computational or dry-lab methods are indispensable to the derivation of novel biological insights (Editors of Nature Methods, 2021). In RNA research, computational or dry-lab methods have been shown to significantly improve the efficiency in shortlisting candidate miRNA sponges for subsequent wet-lab experiments (Le *et al.*, 2017; Li *et al.*, 2018; List *et al.*, 2019; Zhang *et al.*, 2020, 2021b, 2022). To simplify the procedure of exploring miRNA sponge regulation, the miRspongeR package was first published in 2019 (Zhang *et al.*, 2019). Since then, several other *in silico* tools have also been presented, e.g. SPONGE (List *et al.*, 2019), miRSM (Zhang *et al.*, 2021a), CeRNASeek (Zhang *et al.*, 2021b), CeNet Omnibus (Wen *et al.*, 2021) and Crinet (Kesimoglu and Bozdag, 2021). However, until now, all of the existing tools only support the investigation of miRNA sponge regulation regarding a group of samples, rather than the miRNA sponge regulation specific to individual samples. This may have neglected the heterogeneity of miRNA sponge regulation across individual samples. Meanwhile, the development of single-cell and spatial sequencing technology has also necessitated novel tools for exploring miRNA sponge regulation at the resolution of individual samples. Moreover, the identification of miRNA sponge regulation is often computationlly intensive. Nevertheless, most existing tools do not allow parallel computing for the fast identification of miRNA sponge regulation. Additionally, since sample-sample correlation networks could help sample clustering analysis, it is necessary to construct them in the studies of miRNA sponge regulation. To meet these requirements, we present miRspongeR 2.0, an updated and enhanced version of miRspongeR (Zhang *et al.*, 2019) to provide better support for the research on miRNA sponges with higher resolution and more utilities. The updated features of miRspongeR 2.0 can be seen in Table S1 of Supplementary Material S1.

## 2. Implementation

**Fig.1.**
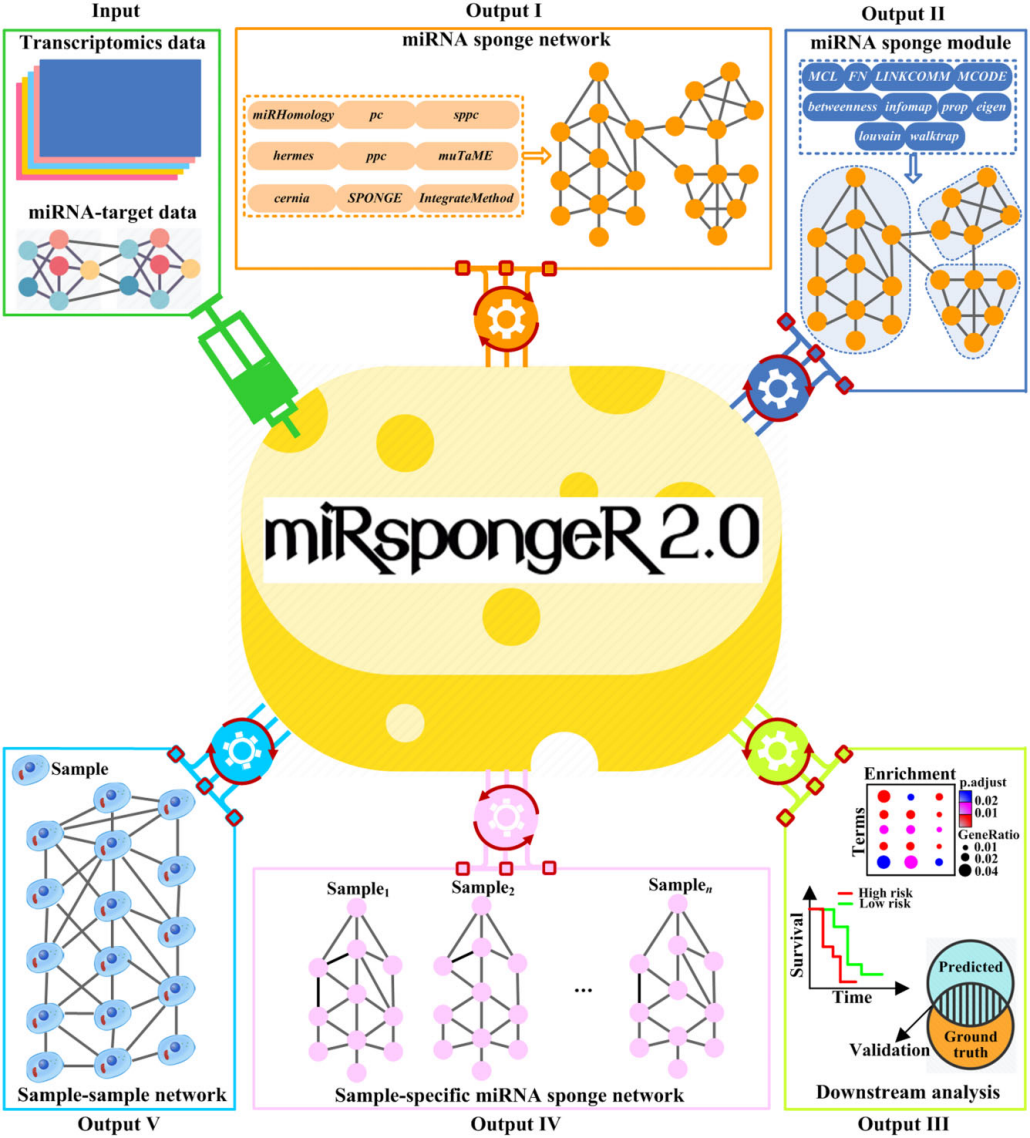
Schema describing input data and utilities for exploring miRNA sponge regulation

As shown in Figure 1, miRspongeR 2.0 implements several utilities to explore miRNA sponge regulation with putative miRNA–target data (including miRNA–target interactions and MREs information) or/and transcriptomics data (including bulk, single-cell and spatial gene expression data).

Firstly, miRspongeR 2.0 provides nine methods for identifying miRNA sponge networks, and 10 methods for inferring miRNA sponge modules. Secondly, miRspongeR 2.0 can infer the miRNA sponge networks for a single sample of interest through a differential miRNA sponge regulation analysis, based on two miRNA sponge networks, one identified using all samples and the other using all samples excluding the sample of interest. Specifically, miRspongeR 2.0 performs a differential analysis between the miRNA sponge network inferred from all samples and the miRNA sponge network inferred from all samples but the sample of interest, and the differential network (i.e. the rewiring of miRNA sponge interactions between the two miRNA sponge networks) is regarded as the miRNA sponge network for the sample of interest. Thirdly, miRspongeR 2.0 implements three methods to construct sample–sample correlation networks based on the identified sample-specific miRNA sponge networks. Finally, miRspongeR 2.0 provides three types of downstream analysis, including enrichment analysis, survival analysis and validation analysis, to discover miRNA sponges with biological implications.

To show the usages of miRspongeR 2.0, we conduct a case study of exploring miRNA sponge regulation by integrating putative miRNA–target interactions and single-cell transcriptomics data. miRspongeR 2.0 allows parallel computation using either computer clusters or personal computers with multi-core CPUs, and users only need to specify the number of cores available for use. For example, our case study has shown that using a personal computer with a 6-core CPU, the runtime of the SPONGE method for identifying a multi-cell miRNA sponge network has been reduced by 1.66 times. The detailed information of this case study is shown in Supplementary Material S1.

## 3. Conclusions

Along with the advancement of single-cell and spatial sequencing technology, miRspongeR 2.0 has significantly enhanced the utilities of the original version introduced in 2019. In miRspongeR 2.0, the new analysis methods like sample-specific miRNA sponge network identification and sample-sample correlation network identification will allow exploring miRNA sponge regulation to a higher resolution and help understand the relationships between samples, respectively. In addition, parallel computation contributes to the fast identification of miRNA sponge regulation and hence enables the application of the computational methods to larger-scale datasets for exploring miRNA sponge regulation. With these new features, we believe that miRspongeR 2.0 will be a useful tool for biologists and bioinformaticians to explore miRNA sponge regulation and conduct subsequent wet-lab experiments.

## Funding

This work was supported by the National Natural Science Foundation of China [61963001]; the Yunnan Fundamental Research Projects [202001AT070024]; and the ARC DECRA [DE200100200].

*Conflict of Interest*: none declared.

## Supplementary Material

vbac063_Supplementary_Data
