# Supplementary material for: miRspongeR 2.0: an enhanced R package for exploring miRNA sponge regulation
Source: Bioinform Adv. 2022 Sep 2;2(1):vbac063. doi: 10.1093/bioadv/vbac063 (PMC9710667; doi:10.1093/bioadv/vbac063)
Supplement: vbac063_Supplementary_Data [file vbac063_supplementary_data.docx]

miRspongeR 2.0: an enhanced R package for exploring miRNA sponge regulation

Junpeng Zhang^1,*^, Lin Liu^2^, Wu Zhang^3^, Xiaomei Li^2^, Chunwen Zhao^1^, Sijing Li^1^, Jiuyong Li^2^ and Thuc Duy Le^2,*^

^1^School of Engineering, Dali University, Dali, China, ^2^UniSA STEM, University of South Australia, Mawson Lakes, SA, Australia, ^3^School of Agriculture and Biological Sciences, Dali University, Dali, China

^*^Corresponding authors: [**zhangjunpeng411@gmail.com**](mailto:zhangjunpeng411@gmail.com)**, thuc.le@unisa.edu.au**

**Table of Contents**

[1 Introduction 1](#_Toc102462687)

[2 Data preparation 2](#_Toc102462688)

[3 Exploring miRNA sponge regulation at the multi-cell level 3](#_Toc102462689)

[4 Exploring miRNA sponge regulation at the single-cell level 6](#_Toc102462690)

[5 Constructing cell-cell correlation networks 11](#_Toc102462691)

[6 Main R scripts 12](#_Toc102462692)

[References 14](#_Toc102462693)

1 Introduction

miRspongeR 2.0 has been released under the GPL-3.0 License, and is available at <http://bioconductor.org/packages/miRspongeR/>. The user manual of miRspongeR 2.0 provides examples illustrating the use of the utility functions at <http://bioconductor.org/packages/devel/bioc/vignettes/miRspongeR/inst/doc/miRspongeR.html>. For user's convenience, we set the parameters of the utility functions their default values to explore miRNA sponge regulation. Compared with miRspongeR 1.0, the updated features of miRspongeR 2.0 are shown in Table S1.

In this supplementary material, to help users study miRNA sponge regulation with their own datasets by using miRspongeR 2.0, we provide a case study on exploring miRNA sponge regulation with single-cell miRNA-mRNA co-sequencing data. In this case study, we infer and analyse miRNA sponge regulation at both the multi-cell and single-cell levels.

**Table S1.** Updated features of miRspongeR 2.0 compared with miRspongeR 1.0.

| **Features** | **miRspongeR 1.0** | **miRspongeR 2.0** |
| --- | --- | --- |
| Acceptable data | Putative miRNA-target data (including miRNA-target interactions and MREs information), bulk transcriptomics data | Putative miRNA-target data (including miRNA-target interactions and MREs information), multiple transcriptomics data (including bulk, single-cell and spatial transcriptomics data) |
| miRNA sponge network | 7 methods with non-parallel computation, and an integrative method | 8 methods with parallel and non-parallel computation, and an integrative method |
| miRNA sponge module | 4 network clustering methods | 10 network clustering methods |
| Sample-specific miRNA sponge network | - | 1 method by using differential miRNA sponge regulation analysis between all samples and all samples without a sample of interest |
| Sample-sample network | - | 3 network similarity methods |
| Downstream analysis | Enrichment analysis, survival analysis, and validation analysis | Enrichment analysis, survival analysis, and validation analysis with expanded ground truth |

2 Data preparation

The single-cell miRNA-mRNA co-sequencing data in 19 half K562 cells (Wang *et al*., 2019) are obtained from Gene Expression Omnibus (GEO, <https://www.ncbi.nlm.nih.gov/geo/>) with accession number GSE114071. Here, the K562 cells are the first human chronic myelogenous leukemia (CML) cell line. For the duplicate miRNAs or mRNAs with the same gene symbols in HGNC (HUGO Gene Nomenclature Committee) but different transcript IDs, we calculate their average expression values as their final expression values. As a feature selection, all of the miRNAs and mRNAs with constant expression values across the 19 half K562 cells are removed. The single-cell miRNA-mRNA co-sequencing data are then pre-processed by using $\log_{2}(x+1)$ transformation. In this case study, we are only interested in CML-related miRNAs and mRNAs included in the single-cell miRNA-mRNA co-sequencing data. The CML-related miRNAs and mRNAs are obtained from HMDD v3.2 (Huang *et al*., 2019) and DisGeNET v7.0 (Piñero *et al*., 2020), respectively. Finally, we have the matched expression data of 59 miRNAs and 3476 mRNAs in the 19 half K562 cells for exploring mRNA-related miRNA sponge regulation.

The putative miRNA-mRNA interactions are from miRTarBase v9.0 (Huang *et al.*, 2020) and TarBase v8.0 (Karagkouni *et al.*, 2018). In miRspongeR 2.0, the built-in ground truth of miRNA sponge interactions is obtained by combining the interactions from three databases including miRSponge (Wang *et al*., 2015), LncCeRBase (Pian *et al.*, 2019) and LncACTdb v3.0 (Wang *et al*., 2022). In total, we have obtained a list of 4029 unique experimentally validated miRNA sponge interactions (including lncRNA, pseudogene, circRNA, and mRNA related miRNA sponge interactions) as the ground-truth for validation.

For convenience, the input data (including single-cell miRNA-mRNA co-sequencing data, and putative miRNA-mRNA interactions) used in this case study can be obtained from <https://github.com/zhangjunpeng411/K562_CML>.

3 Exploring miRNA sponge regulation at the multi-cell level

At the network layer, miRspongeR 2.0 provides eight popular methods, including miRHomology (Li *et al*., 2014; Sarver and Subramanian, 2012), pc (Zhou *et al*., 2014; Xu *et al*., 2015), sppc (Paci *et al*., 2014), ppc (Le *et al*., 2017), hermes (Sumazin *et al*., 2011), muTaME (Tay *et al*., 2011), cernia (Sardina *et al*., 2017), and SPONGE (List *et al*., 2019), to infer miRNA sponge networks. In this case study, we choose the latest method SPONGE to identify miRNA sponge network from putative miRNA-mRNA interactions and single-cell transcriptomics data. For SPONGE, the cutoff of shared miRNAs, adjusted *p*-value and sensitivity correlation is set to 1, 0.05 and 0.10, respectively. The number of CPU cores is set to 6, and the number of datasets sampled is set to 100 for the null model. As a result, we can construct a miRNA sponge network (including 352 nodes and 355 edges) at the multi-cell level (see Figure S1). By using the *NetworkAnalyzer* plugin (Assenov *et al*., 2008) in Cytoscape (Shannon *et al*., 2003), the distribution of node degrees of the miRNA sponge network follows power law distribution with *R*^2^=0.847 (in the form of *y*=376.71*x*^-2.446^). Here, the *R*^2^ value is a deterministic coefficient to measure the quality of a power curve fit. The interval of *R*^2^ value is [0 1]. A larger *R*^2^ value indicates a better power law curve fit. Therefore, this result indicates that the identified miRNA sponge network is approximately scale-free. Since the ground truth of miRNA sponge regulation is still limited, in this case study, the number of validated miRNA sponge interactions is 0.


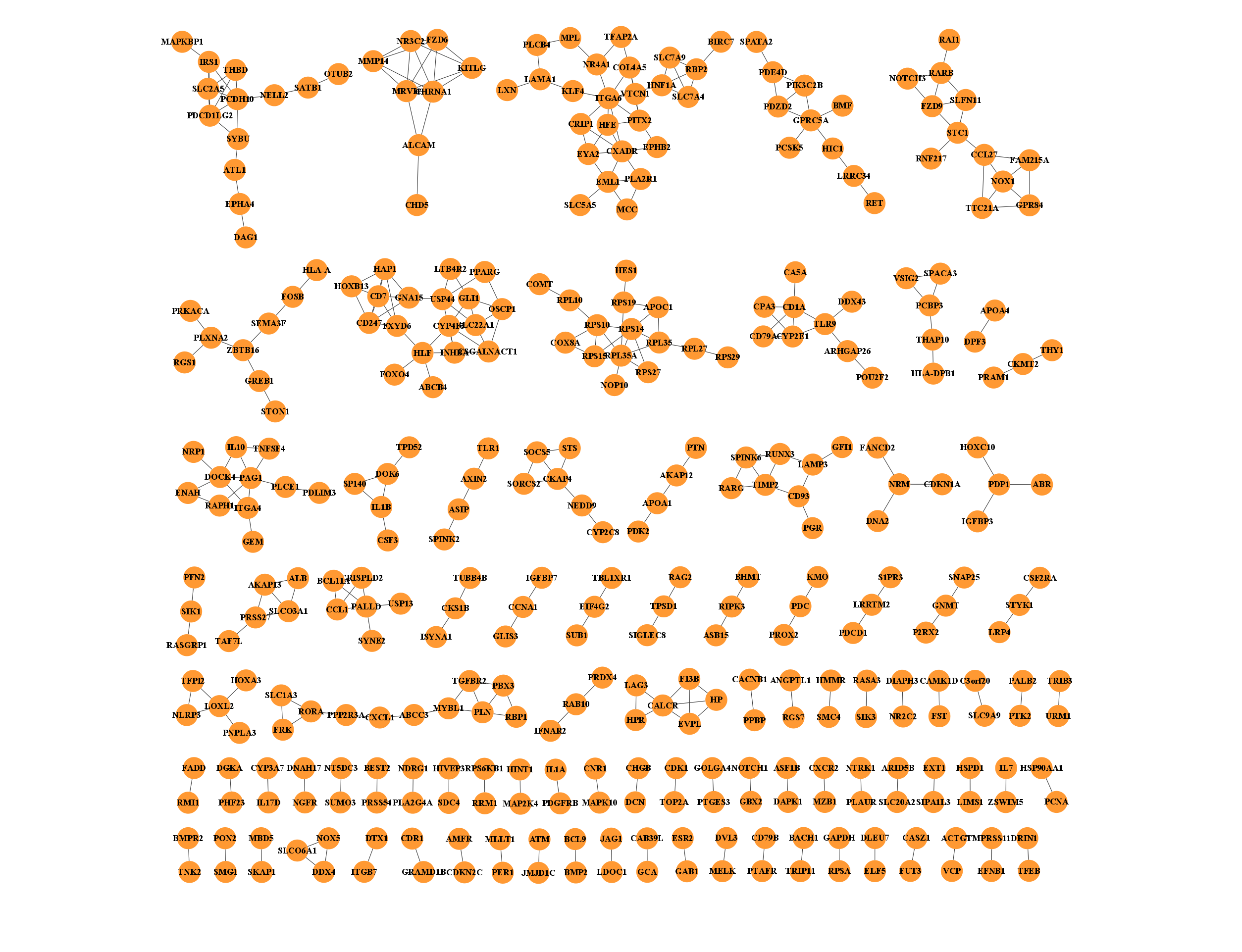


**Fig. S1.** Network visualization of the identified miRNA sponge network.

At the module layer, miRspongeR 2.0 provides 10 popular network clustering methods, including FN (Clauset *et al*., 2004), MCL (Enright *et al*., 2002), LINKCOMM (Kalinka and Tomancak, 2011), MCODE (Bader and Hogue, 2003), betweenness (Newman and Girvan, 2004), infomap (Rosvall and Bergstrom, 2008), prop (Raghavan *et al*., 2007), eigen (Newman, 2006), louvain (Blondel *et al*., 2008), and walktrap (Pons and Latapy, 2005), to identify miRNA sponge modules. In this case study, we select the default method MCL (frequently used for network clustering) to identify miRNA sponge modules from the identified miRNA sponge network. The number of miRNA sponges included in each module is at least 5. In total, we have obtained 20 miRNA sponge modules (see Table S2). We further conduct enrichment analysis (including disease and functional enrichment analysis) of the identified miRNA sponge modules. The disease databases used include Disease Ontology (DO, http://disease-ontology.org/), DisGeNET (DGN, http://www.disgenet.org/) and Network of Cancer Genes (NCG, http://ncg.kcl.ac.uk/). The ontology databases used are Gene Ontology (GO, http:// www.geneontology.org/), Kyoto Encyclopedia of Genes and Genomes (KEGG, http://www.genome.jp/kegg/), and Reactome (http://reactome.org/). Enrichment analysis shows that all of the identified 20 miRNA sponge modules are enriched in at least one of GO, KEGG, Reactome, DO, DisGeNET, and NCG terms (see Table S3). This result displays that all of miRNA sponge modules are functional.

**Table S2.** The identified 20 miRNA sponge modules.

| **Module ID** | **Size** | **miRNA sponges** |
| --- | --- | --- |
| 1 | 5 | AKAP13, ALB, PRSS27, SLCO3A1, TAF7L |
| 2 | 6 | CALCR, EVPL, F13B, HPR, HP, LAG3 |
| 3 | 5 | CA5A, CD1A, CD79A, CPA3, CYP2E1 |
| 4 | 6 | CD247, CD7, FXYD6, GNA15, HAP1, HOXB13 |
| 5 | 6 | CHRNA1, FZD6, KITLG, MMP14, MRVI1, NR3C2 |
| 6 | 7 | BMF, GPRC5A, PDE4D, PDZD2, PCSK5, PIK3C2B, SPATA2 |
| 7 | 5 | ABCB4, CYP4F3, FOXO4, HLF, INHBA |
| 8 | 5 | CSF3, DOK6, IL1B, SP140, TPD52 |
| 9 | 7 | COL4A5, EPHB2, HFE, ITGA6, PITX2, TFAP2A, VTCN1 |
| 10 | 5 | KLF4, LAMA1, MPL, LXN, PLCB4 |
| 11 | 5 | HOXA3, LOXL2, NLRP3, PNPLA3, TFPI2 |
| 12 | 5 | CCL27, FAM215A, GPR84, NOX1, TTC21A |
| 13 | 9 | DOCK4, ENAH, GEM, IL10, ITGA4, PAG1, NRP1, RAPH1, TNFSF4 |
| 14 | 6 | BCL11A, CCL1, CRISPLD2, PALLD, SYNE2, USP13 |
| 15 | 7 | IRS1, PCDH10, PDCD1LG2, SLC2A5, SYBU, MAPKBP1, THBD |
| 16 | 5 | MYBL1, PBX3, PLN, TGFBR2, RBP1 |
| 17 | 5 | FZD9, NOTCH3, RAI1, RARB, SLFN11 |
| 18 | 5 | BIRC7, HNF1A, RBP2, SLC7A4, SLC7A9 |
| 19 | 6 | APOC1, NOP10, RPL35, RPL35A, RPS14, RPS27 |
| 20 | 7 | CSGALNACT1, GLI1, LTB4R2, OSCP1, PPARG, SLC22A1, USP44 |

**Table S3.** Enrichment analysis of the identified miRNA sponge modules.

| **Module ID** | **#GO terms** | **#KEGG terms** | **#Reactome terms** | **#DO terms** | **#DisGeNET terms** | **#NCG terms** |
| --- | --- | --- | --- | --- | --- | --- |
| 1 | 0 | 0 | 19 | 0 | 480 | 0 |
| 2 | 2 | 0 | 4 | 3 | 235 | 0 |
| 3 | 0 | 0 | 8 | 8 | 144 | 0 |
| 4 | 0 | 1 | 38 | 0 | 0 | 0 |
| 5 | 50 | 1 | 0 | 14 | 235 | 1 |
| 6 | 1 | 0 | 4 | 0 | 0 | 0 |
| 7 | 0 | 0 | 16 | 0 | 83 | 0 |
| 8 | 0 | 5 | 15 | 0 | 1291 | 0 |
| 9 | 112 | 4 | 22 | 2 | 393 | 0 |
| 10 | 68 | 0 | 18 | 0 | 98 | 0 |
| 11 | 0 | 0 | 9 | 0 | 358 | 2 |
| 12 | 0 | 0 | 4 | 3 | 0 | 0 |
| 13 | 330 | 3 | 0 | 0 | 738 | 0 |
| 14 | 0 | 3 | 0 | 0 | 55 | 0 |
| 15 | 0 | 0 | 15 | 0 | 3 | 2 |
| 16 | 98 | 2 | 13 | 3 | 159 | 0 |
| 17 | 62 | 3 | 8 | 0 | 185 | 6 |
| 18 | 8 | 3 | 10 | 0 | 132 | 1 |
| 19 | 88 | 3 | 37 | 0 | 104 | 0 |
| 20 | 1 | 0 | 0 | 0 | 211 | 0 |

4 Exploring miRNA sponge regulation at the single-cell level

By simulating the single-intervention experiments (e.g. knocking down a cell each time), miRspongeR 2.0 can explore miRNA sponge regulation at the resolution of individual cells.

At the network layer, miRspongeR 2.0 infers the miRNA sponge networks for a single cell of interest through a differential miRNA sponge regulation analysis, based on two miRNA sponge networks, one identified using all cells and the other using all cells excluding the cell of interest. Specifically, miRspongeR 2.0 performs a differential analysis between the miRNA sponge network inferred from all cells and the miRNA sponge network inferred from all cells but the cell of interest, and the differential network (i.e. the rewiring of miRNA sponge interactions between the two miRNA sponge networks) is regarded as the miRNA sponge network for the cell of interest. In this case study, the two miRNA sponge networks are also identified by using SPONGE. For SPONGE, the cutoff of shared miRNAs, adjusted *p*-value and sensitivity correlation is set to 1, 0.05 and 0.10, respectively. The number of CPU cores is set to 6, and the number of datasets sampled is set to 100 for the null model. As a result, we have identified 19 cell-specific miRNA sponge networks at the single-cell level (see Table S4). By using the *NetworkAnalyzer* plugin in Cytoscape, the distributions of node degrees of the identified 19 cell-specific miRNA sponge networks follow power law distribution with *R*^2^>0.77. This result reveals that the identified 19 cell-specific miRNA sponge networks are all approximately scale-free. Since the ground truth of miRNA sponge regulation is also limited, the number of validated miRNA sponge interactions for each cell is 0.

At the module layer, we also select the default method MCL (frequently used for network clustering) to identify cell-specific miRNA sponge modules from the identified cell-specific miRNA sponge networks. The number of miRNA sponges included in each module is at least 5. As shown in Table S5, the identified cell-specific miRNA sponge modules are considerably different for each single cell. We also conduct enrichment analysis (including disease and functional enrichment analysis) of the identified cell-specific miRNA sponge modules. Enrichment analysis indicates that all of the identified cell-specific miRNA sponge modules are enriched in at least one of GO, KEGG, Reactome, DO, DisGeNET, and NCG terms (see Table S6). This result reveals that all of cell-specific miRNA sponge modules are functional.

**Table S4.** The identified 19 cell-specific miRNA sponge networks.

| **Single-Cell ID** | **Nodes** | **Edges** | ***y*=*bx^a^*** | ***R*^2^** |
| --- | --- | --- | --- | --- |
| K562_HalfCell_01 | 125 | 96 | *y*=65.208*x^-^*^2.023^ | 0.886 |
| K562_HalfCell_02 | 118 | 71 | *y*=109.63*x^-^*^3.085^ | 0.972 |
| K562_HalfCell_03 | 137 | 90 | *y*=67.759*x^-^*^2.401^ | 0.864 |
| K562_HalfCell_04 | 109 | 81 | *y*=68.994*x^-^*^2.328^ | 0.881 |
| K562_HalfCell_05 | 107 | 69 | *y*=46.685*x^-^*^2.157^ | 0.847 |
| K562_HalfCell_07 | 162 | 118 | *y*=81.362*x^-^*^2.070^ | 0.885 |
| K562_HalfCell_08 | 122 | 87 | *y*=90.825*x^-^*^2.594^ | 0.930 |
| K562_HalfCell_09 | 152 | 129 | *y*=88.675*x^-^*^1.985^ | 0.985 |
| K562_HalfCell_10 | 134 | 97 | *y*=82.417*x^-^*^2.325^ | 0.969 |
| K562_HalfCell_11 | 127 | 83 | *y*=107.22*x^-^*^2.796^ | 0.985 |
| K562_HalfCell_12 | 151 | 122 | *y*=87.391*x^-^*^1.972^ | 0.954 |
| K562_HalfCell_13 | 116 | 78 | *y*=58.512*x^-^*^2.241^ | 0.818 |
| K562_HalfCell_14 | 157 | 110 | *y*=111.612*x^-^*^2.449^ | 0.957 |
| K562_HalfCell_15 | 136 | 98 | *y*=61.855*x^-^*^1.964^ | 0.778 |
| K562_HalfCell_16 | 142 | 95 | *y*=94.195*x^-^*^2.485^ | 0.948 |
| K562_HalfCell_17 | 134 | 87 | *y*=92.727*x^-^*^2.536^ | 0.976 |
| K562_HalfCell_18 | 125 | 85 | *y*=110.05*x^-^*^2.826^ | 0.951 |
| K562_HalfCell_19 | 130 | 84 | *y*=73.169 *x^-^*^2.426^ | 0.913 |
| K562_HalfCell_20 | 130 | 79 | *y*=108.94 *x^-^*^3.090^ | 0.984 |

**Table S5.** The identified cell-specific miRNA sponge modules.

| **Single-cell ID** | **Module ID** | **Size** | **miRNA sponges** |
| --- | --- | --- | --- |
| K562_HalfCell_01 | 1 | 6 | DOCK4, PAG1, NRP1, ITGA4, IL10, ENAH |
|  | 2 | 11 | MMP14, KITLG, FZD6, GPRC5A, BMF, MRVI1, CHRNA1, MYBL1, PIK3C2B, NR3C2, PDE4D |
| K562_HalfCell_02 | / | / | / |
| K562_HalfCell_03 | 1 | 9 | TPSD1, CYP2E1, CPA3, CD79A, CD1A, CA5A, TLR9 |
|  | 2 | 6 | DOCK4, PAG1, NRP1, ITGA4, IL10, ENAH |
|  | 3 | 8 | PDP1, IGFBP3, HOXC10, NR3C2, KITLG, FZD6, MRVI1, CHRNA1 |
| K562_HalfCell_04 | 1 | 6 | PBX3, RUNX3, RORA, FRK, ENC1, RBP1 |
|  | 2 | 7 | TGFBR2, PLN, USP44, SLC22A1, INHBA, MYBL1, GNG11 |
|  | 3 | 6 | DOCK4, PAG1, NRP1, ITGA4, IL10, ENAH |
|  | 4 | 8 | HPR, F13B, EVPL, CALCR, PTAFR, CD79B, LAG3, HP |
| K562_HalfCell_05 | 1 | 5 | SLCO6A1, NOX5, DDX4, P2RX2, GNMT |
|  | 2 | 8 | CKAP4, NR3C2, MMP14, CHD5, ALCAM, STS, SORCS2, CNR1 |
| K562_HalfCell_07 | 1 | 5 | RARB, NOTCH3, FZD9, RAI1, SLFN11 |
|  | 2 | 6 | DOCK4, PAG1, NRP1, ITGA4, IL10, ENAH |
|  | 3 | 10 | STC1, RNF217, KLF4, MCC, HFE, EPHB2, EML1, CXADR, PITX2, ITGA6 |
|  | 4 | 10 | TTC21A, NOX1, FAM215A, CCL27, HP F13B, EVPL, CALCR, GPR84, PRAM1 |
| K562_HalfCell_08 | 1 | 10 | TNFSF4, PAG1, ITGA4, IL10, ENAH, DOCK4, RAPH1, GEM, TIMP3, NRP1 |
|  | 2 | 5 | SLFN11, FZD9, GRAMD1B, CDR1, TRIB1 |
|  | 3 | 6 | GPR84, TTC21A, NOX1, FAM215A, CCL27, ASB2 |
| K562_HalfCell_09 | 1 | 7 | NR4A1, SLFN11, FZD9, TFAP2A, GRAMD1B, MPL, FOXP2 |
|  | 2 | 6 | DOCK4, PAG1, NRP1, ITGA4, IL10, ENAH |
|  | 3 | 12 | PLA2R1, EML1, EYA2, CXADR, CRIP1, HP, MYBL1, MCC, SLC5A5, TGFBR2, PLN, EPHB2 |
|  | 4 | 5 | PITX2, ITGA6, COL4A5, VTCN1, NOTCH3 |
|  | 5 | 5 | SLC7A4, HNF1A, RBP2, BIRC7, SLC7A9 |
|  | 6 | 7 | NR3C2, MMP14, KITLG, FZD6, MRVI1, CHRNA1, HFE |
| K562_HalfCell_10 | 1 | 5 | TRIM62, ABCB1, CCNA1, FST, FSD1 |
|  | 2 | 6 | HIC1, LAMP3, CD93, IL6R, SPINK6, RUNX3 |
|  | 3 | 6 | DOCK4, PAG1, NRP1, ITGA4, IL10, ENAH |
|  | 4 | 7 | PDE4D, PIK3C2B, PDZD2, GPRC5A, BMF, SPATA2, PCSK5 |
|  | 5 | 6 | RBP2, APOA4, SLC7A9, SLC7A4, HNF1A, BIRC7 |
|  | 6 | 8 | DNAH17, CYP2E1, CPA3, CD79A, CD1A, CA5A, TLR9, NGFR |
| K562_HalfCell_11 | 1 | 5 | PDLIM3, PLCE1, TNFSF4, PAG1, LRIG1 |
|  | 2 | 5 | KITLG, MRVI1, CHRNA1, ALCAM, SLC30A8 |
| K562_HalfCell_12 | 1 | 7 | TCF7L1, MCC, PLA2R1, EML1, EYA2, CXADR, SCD5 |
|  | 2 | 5 | MMP9, ABTB2, SLC1A3, FRK, ENC1 |
|  | 3 | 5 | DOCK4, PAG1, ITGA4, IL10, ENAH |
|  | 4 | 12 | GLI1, SLC22A1, OSCP1, INHBA, CYP4F3, CSGALNACT1, NR3C2, MMP14, KITLG, FZD6, MRVI1, CHRNA1 |
|  | 5 | 6 | HAP1, GNA15, FXYD6, CD7, CD247, HOXB13 |
| K562_HalfCell_13 | 1 | 5 | GNMT, NOX5, DDX4, SNAP25, P2RX2 |
|  | 2 | 7 | RORA, FRK, STS, SOCS5, NEDD9, CKAP4, SLC1A3 |
|  | 3 | 6 | DOCK4, PAG1, NRP1, ITGA4, IL10, ENAH |
| K562_HalfCell_14 | 1 | 5 | TPSD1, RAG2, PRAM1, CKMT2, SIGLEC8 |
|  | 2 | 5 | NLRP3, LOXL2, HOXA3, TFPI2, PNPLA3 |
|  | 3 | 5 | EIF4G2, PSMD12, EIF4E, TBL1XR1, SUB1 |
|  | 4 | 6 | DOCK4, PAG1, NRP1, ITGA4, IL10, ENAH |
|  | 5 | 5 | CPA3, CD79A, CD1A, TLR9, CYP2E1 |
|  | 6 | 7 | CA5A, SCD5, KLF4, SLC7A9, SLC7A4, HFE, ITGA6 |
|  | 7 | 5 | SLFN11, NOTCH3, FZD9, STC1, TKTL1 |
| K562_HalfCell_15 | 1 | 5 | PTPRU, ACE, MPL, PLCB4, ADCY9 |
|  | 2 | 7 | OTUB2, SATB1, NELL2, EYA2, CRIP1, TACC2, EML6 |
|  | 3 | 9 | THBD, SLC2A5, PDCD1LG2, PCDH10, IRS1, MST1R, PLAT, PLXNA2, MAPKBP1 |
|  | 4 | 5 | DOCK4, NRP1, ITGA4, IL10, ENAH |
| K562_HalfCell_16 | 1 | 10 | RARG, RUNX3, PIK3C2B, PDZD2, HIC1, EFNB1, DNAH17, TIMP2, BMF, SPINK6 |
|  | 2 | 6 | DOCK4, PAG1, NRP1, ITGA4, IL10, ENAH |
|  | 3 | 5 | MZB1, CXCR2, STS, SLCO6A1, DDX4 |
| K562_HalfCell_17 | 1 | 6 | DOCK4, PAG1, NRP1, ITGA4, IL10, ENAH |
|  | 2 | 7 | PALLD, CRISPLD2, CCL1, BCL11A, SPINK6, USP13, TIMP2 |
|  | 3 | 6 | AXIN2, SLFN11, NOTCH3, GRAMD1B, STC1, TLR1 |
| K562_HalfCell_18 | 1 | 8 | GEM, PAG1, ITGA4, ENAH, DOCK4, PTX3, NRP1, IL10 |
|  | 2 | 5 | FST, CAMK1D, TFPI2, NLRP3, LOXL2 |
|  | 3 | 5 | GLI1, CYP4F3, HLF, ABCB4, ABCB1 |
| K562_HalfCell_19 | 1 | 7 | SVIL, PDZD2, HIC1, DNAH17, GPRC5A, BMF, CDA |
|  | 2 | 5 | GRIA3, INHBA, RGS7, ANGPTL1, GPR37 |
|  | 3 | 5 | DOK6, IL1B, CSF3, TPD52, SP140 |
|  | 4 | 6 | DOCK4, PAG1, NRP1, ITGA4, IL10, ENAH |
| K562_HalfCell_20 | 1 | 6 | DOCK4, PAG1, NRP1, ITGA4, IL10, ENAH |
|  | 2 | 6 | PRSS27, ALB, AKAP13, TAF7L, LTB4R2, SLCO3A1 |

**Table S6.** Enrichment analysis of the identified cell-specific miRNA sponge modules.

| **Single-cell ID** | **Module ID** | **#GO terms** | **#KEGG terms** | **#Reactome terms** | **#DO terms** | **#DisGeNET terms** | **#NCG terms** |
| --- | --- | --- | --- | --- | --- | --- | --- |
| K562_HalfCell_01 | 1 | 265 | 6 | 0 | 0 | 901 | 0 |
|  | 2 | 0 | 0 | 0 | 0 | 1 | 0 |
| K562_HalfCell_02 | / | / | / | / | / | / | / |
| K562_HalfCell_03 | 1 | 8 | 1 | 0 | 0 | 223 | 0 |
|  | 2 | 265 | 6 | 0 | 0 | 901 | 0 |
|  | 3 | 0 | 1 | 0 | 21 | 3 | 1 |
| K562_HalfCell_04 | 1 | 5 | 0 | 7 | 0 | 11 | 0 |
|  | 2 | 6 | 0 | 36 | 1 | 74 | 0 |
|  | 3 | 265 | 6 | 0 | 0 | 901 | 0 |
|  | 4 | 0 | 0 | 2 | 5 | 10 | 0 |
| K562_HalfCell_05 | 1 | 0 | 2 | 6 | 3 | 87 | 0 |
|  | 2 | 0 | 0 | 0 | 11 | 290 | 1 |
| K562_HalfCell_07 | 1 | 62 | 3 | 8 | 0 | 185 | 6 |
|  | 2 | 265 | 6 | 0 | 0 | 901 | 0 |
|  | 3 | 22 | 0 | 0 | 0 | 303 | 0 |
|  | 4 | 0 | 1 | 11 | 0 | 1 | 0 |
| K562_HalfCell_08 | 1 | 300 | 2 | 0 | 0 | 763 | 0 |
|  | 2 | 59 | 12 | 5 | 0 | 32 | 0 |
|  | 3 | 0 | 0 | 4 | 3 | 0 | 0 |
| K562_HalfCell_09 | 1 | 12 | 1 | 8 | 0 | 99 | 2 |
|  | 2 | 265 | 6 | 0 | 0 | 901 | 0 |
|  | 3 | 2 | 0 | 0 | 0 | 41 | 0 |
|  | 4 | 18 | 5 | 27 | 2 | 288 | 6 |
|  | 5 | 8 | 3 | 10 | 0 | 132 | 1 |
|  | 6 | 11 | 1 | 0 | 26 | 297 | 1 |
| K562_HalfCell_10 | 1 | 0 | 0 | 0 | 0 | 421 | 2 |
|  | 2 | 49 | 0 | 6 | 0 | 3 | 0 |
|  | 3 | 265 | 6 | 0 | 0 | 901 | 0 |
|  | 4 | 1 | 0 | 4 | 0 | 0 | 0 |
|  | 5 | 74 | 1 | 17 | 0 | 128 | 1 |
|  | 6 | 0 | 0 | 14 | 0 | 210 | 0 |
| K562_HalfCell_11 | 1 | 116 | 0 | 10 | 0 | 0 | 1 |
|  | 2 | 0 | 0 | 9 | 0 | 193 | 0 |
| K562_HalfCell_12 | 1 | 0 | 1 | 10 | 0 | 4 | 1 |
|  | 2 | 0 | 0 | 0 | 2 | 499 | 1 |
|  | 3 | 235 | 5 | 1 | 0 | 958 | 0 |
|  | 4 | 50 | 0 | 0 | 0 | 28 | 0 |
|  | 5 | 0 | 1 | 38 | 0 | 0 | 0 |
| K562_HalfCell_13 | 1 | 0 | 4 | 16 | 0 | 124 | 0 |
|  | 2 | 93 | 0 | 0 | 1 | 135 | 1 |
|  | 3 | 265 | 6 | 0 | 0 | 901 | 0 |
| K562_HalfCell_14 | 1 | 0 | 2 | 2 | 2 | 72 | 0 |
|  | 2 | 0 | 0 | 9 | 0 | 358 | 2 |
|  | 3 | 0 | 0 | 3 | 0 | 157 | 4 |
|  | 4 | 265 | 6 | 0 | 0 | 901 | 0 |
|  | 5 | 59 | 0 | 14 | 0 | 315 | 0 |
|  | 6 | 0 | 0 | 13 | 0 | 1 | 0 |
|  | 7 | 0 | 1 | 12 | 0 | 91 | 2 |
| K562_HalfCell_15 | 1 | 45 | 41 | 26 | 3 | 616 | 0 |
|  | 2 | 25 | 0 | 0 | 0 | 16 | 0 |
|  | 3 | 17 | 0 | 0 | 3 | 308 | 1 |
|  | 4 | 323 | 6 | 0 | 0 | 958 | 2 |
| K562_HalfCell_16 | 1 | 0 | 0 | 0 | 0 | 11 | 0 |
|  | 2 | 265 | 6 | 0 | 0 | 901 | 0 |
|  | 3 | 0 | 0 | 7 | 1 | 328 | 0 |
| K562_HalfCell_17 | 1 | 265 | 6 | 0 | 0 | 901 | 0 |
|  | 2 | 52 | 3 | 0 | 0 | 7 | 1 |
|  | 3 | 3 | 1 | 24 | 0 | 119 | 0 |
| K562_HalfCell_18 | 1 | 171 | 6 | 0 | 2 | 909 | 0 |
|  | 2 | 0 | 0 | 13 | 0 | 359 | 2 |
|  | 3 | 72 | 5 | 6 | 5 | 581 | 0 |
| K562_HalfCell_19 | 1 | 0 | 0 | 5 | 0 | 0 | 1 |
|  | 2 | 61 | 0 | 10 | 0 | 36 | 0 |
|  | 3 | 0 | 5 | 15 | 0 | 1291 | 0 |
|  | 4 | 265 | 6 | 0 | 0 | 901 | 0 |
| K562_HalfCell_20 | 1 | 265 | 6 | 0 | 0 | 901 | 0 |
|  | 2 | 0 | 0 | 9 | 0 | 347 | 0 |

5 Constructing cell-cell correlation networks

The cell-cell correlation networks could help reveal the relationships or communications between single-cells, and contribute to single-cell clustering analysis. Therefore, miRspongeR 2.0 provides three similarity methods including Simpson (Tucker *et al*., 2017), Jaccard (Jaccard, 1912) and Lin (Lin *et al*., 1998), to construct cell-cell correlation networks based on the identified cell-specific miRNA sponge networks. In this case study, we use the default method Simpson (frequently used for calculating network similarity) to construct cell-cell correlation networks and the cutoff of similarity and adjusted *p*-value is set to 0.30 and 0.05, respectively. Therefore, we can infer a cell-cell correlation network consisting of 19 nodes and 122 edges (see Figure S2). By using the *NetworkAnalyzer* plugin in Cytoscape, the distribution of node degrees of the cell-cell correlation network doesn’t follow power law distribution with *R*^2^=0.190 (in the form of *y*=0.795*x*^0.278^). This result shows that the identified cell-cell correlation network is not scale-free.


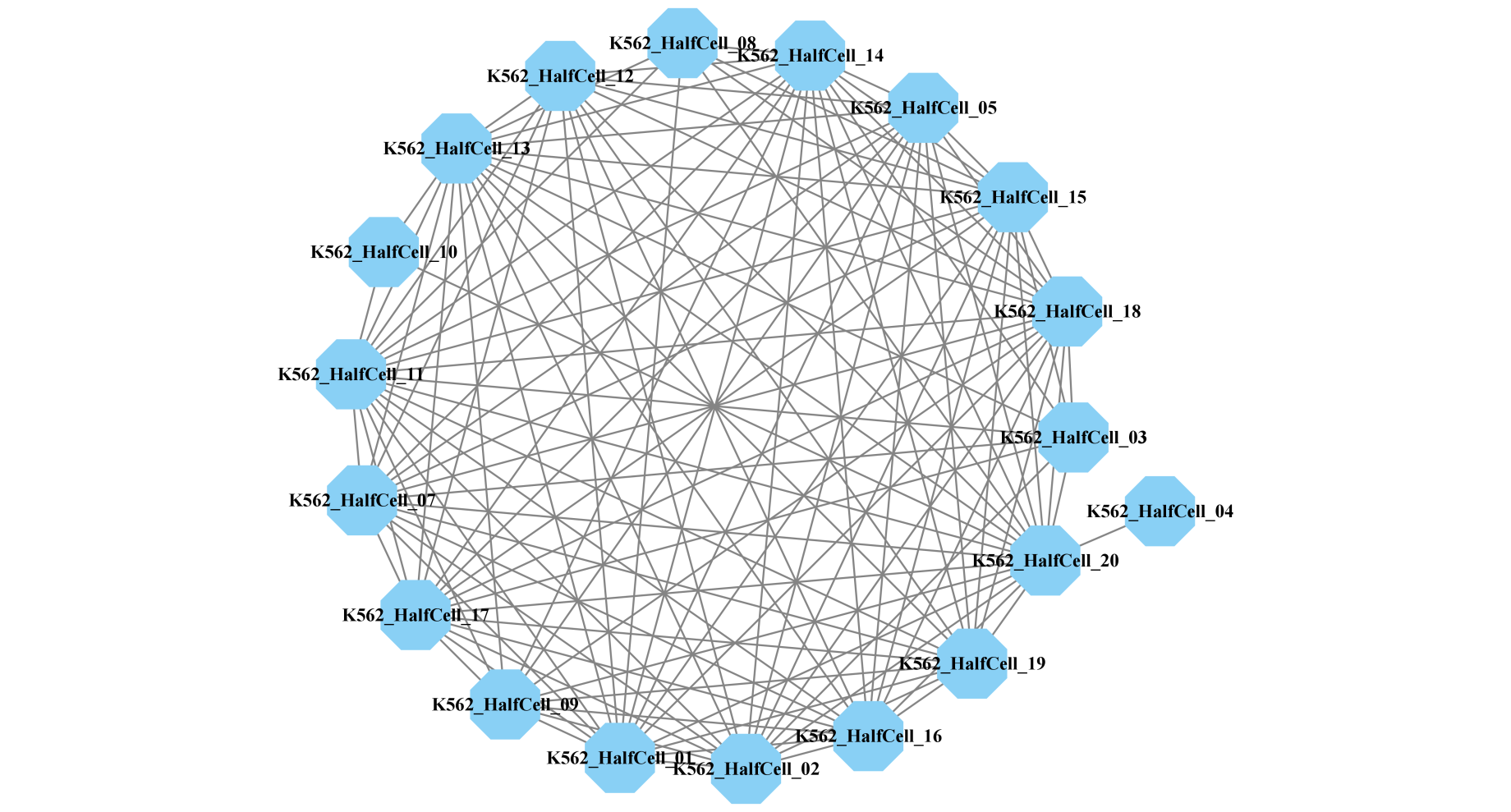


**Fig. S2.** Network visualization of the identified cell-cell correlation network.

6 Main R scripts

## Load required dataset and R packages

load("K562_CML.RData")

library(miRspongeR)

library(SPONGE)

library(doParallel)

library(igraph)

## Constructing null model

pre_null_model <- sponge_build_null_model(number_of_datasets = 100,

number_of_samples = nrow(miRNA_scRNA_CML))

## Inferring miRNA sponge networks

sponge_net <- spongeMethod(miRTarget_CML,

cbind(miRNA_scRNA_CML, mRNA_scRNA_CML),

minSharedmiR = 1,

padjustvaluecutoff = 0.05,

senscorcutoff = 0.1,

null_model = pre_null_model,

method = "sponge_parallel",

num.cores = 6)

## Validation of miRNA sponge networks

Groundtruthcsv <- system.file("extdata", "Groundtruth.csv", package="miRspongeR")

Groundtruth <- read.csv(Groundtruthcsv, header=TRUE, sep=",")

sponge_net_validated <- spongeValidate(sponge_net[, 1:2], directed = FALSE, Groundtruth)

## Inferring miRNA sponge modules

sponge_module <- netModule(sponge_net[, 1:2], modulesize = 5)

## Enrichment analysis of miRNA sponge modules

sponge_module_DEA <- moduleDEA(sponge_module)

sponge_module_FEA <- moduleFEA(sponge_module)

## Inferring cell-specific miRNA sponge networks

sponge_cell_specific_net <- sponge_sample_specific(miRTarget_CML,

cbind(miRNA_scRNA_CML,

mRNA_scRNA_CML),

minSharedmiR = 1,

padjustvaluecutoff = 0.05,

senscorcutoff = 0.1,

null_model = pre_null_model,

method = "sponge_parallel",

num.cores = 6)

## Validation of cell-specific miRNA sponge networks

sponge_cell_specific_net_validated <- lapply(seq(sponge_cell_specific_net),

function(i)

spongeValidate(sponge_cell_specific_net[[i]][, 1:2], directed = FALSE,

Groundtruth))

## Inferring cell-specific miRNA sponge modules

sponge_cell_specific_module <- lapply(seq(sponge_cell_specific_net),

function(i)

netModule(sponge_cell_specific_net[[i]],

modulesize = 5))

## Enrichment analysis of cell-specific miRNA sponge modules

sponge_cell_specific_module_DEA <- lapply(seq(sponge_cell_specific_module),

function(i)

moduleDEA(sponge_cell_specific_module[[i]]))

sponge_cell_specific_module_FEA <- lapply(seq(sponge_cell_specific_module),

function(i)

moduleFEA(sponge_cell_specific_module[[i]]))

## Identifying cell-cell correlation network

cell_cor_net <- sample_cor_network(sponge_cell_specific_net,

genes_num = ncol(mRNA_scRNA_CML),

simcutoff = 0.3,

padjustvaluecutoff = 0.05)

save.image("K562_CML.RData")

References

Assenov, Y. *et al*. (2008) Computing topological parameters of biological networks. *Bioinformatics*, **24**, 282-4.

Bader, G.D. and Hogue, C.W. (2003) An automated method for finding molecular complexes in large protein interaction networks. *BMC Bioinformatics*, **4**, 2.

Blondel, V.D. *et al*. (2008) Fast unfolding of communities in large networks. *Journal of statistical mechanics: theory and experiment*, **2008**, P10008.

Clauset, A. *et al*. (2004) Finding community structure in very large networks. *Phys Rev E Stat Nonlin Soft Matter Phys*., **70**, 066111.

Enright. A.J. (2002) An efficient algorithm for large-scale detection of protein families. *Nucleic Acids Res*., **30**, 1575-84.

Huang, H.Y. *et al*. (2020) miRTarBase 2020: updates to the experimentally validated microRNA-target interaction database. *Nucleic Acids Res*., **48**, D148-D154.

Huang, Z. *et al*. (2019) HMDD v3.0: a database for experimentally supported human microRNA-disease associations. *Nucleic Acids Res*., **47**, D1013-D1017.

Jaccard, P. (1912) The distribution of the flora in the alpine zone. *The New Phytologist*, **11**, 37–50.

Kalinka, A.T. and Tomancak, P. (2011) linkcomm: an R package for the generation, visualization, and analysis of link communities in networks of arbitrary size and type. Bioinformatics, **27**, 2011-2.

Karagkouni, D. *et al*. (2018) DIANA-TarBase v8: a decade-long collection of experimentally supported miRNA-gene interactions. *Nucleic Acids Res*., **46**, D239-D245.

Le, T.D. *et al*. (2017) Computational methods for identifying miRNA sponge interactions. *Brief Bioinform*., **18**, 577-90.

Li, J.H. *et al*. (2014) starBase v2.0: decoding miRNA-ceRNA, miRNA-ncRNA and protein-RNA interaction networks from large-scale CLIP-Seq data. *Nucleic Acids Res*., **42**, D92-7.

Lin, D. *et al*. (1998) An information-theoretic definition of similarity. In: *Proceedings of the Fifteenth International Conference on Machine Learning*, **98**, 296–304.

List, M. *et al*. (2019) Large-scale inference of competing endogenous RNA networks with sparse partial correlation. *Bioinformatics*. **35**, i596-i604.

Newman, M.E. and Girvan, M. (2004) Finding and evaluating community structure in networks. *Phys Rev E Stat Nonlin Soft Matter Phys*. **69**, 026113.

Newman, M.E. (2006) Finding community structure in networks using the eigenvectors of matrices. *Phys Rev E Stat Nonlin Soft Matter Phys*, 74, 036104.

Paci, P. *et al*. (2014) Computational analysis identifies a sponge interaction network between long non-coding RNAs and messenger RNAs in human breast cancer. *BMC Syst Biol*., **8**, 83.

Pian, C. *et al*. (2019) LncCeRBase: a database of experimentally validated human competing endogenous long non-coding RNAs. *Database (Oxford)*, **2019**, baz090.

Piñero, J. *et al*. (2020) The DisGeNET knowledge platform for disease genomics: 2019 update. *Nucleic Acids Res*., **48**, D845-D855.

Pons, P. and Latapy, M. (2005) Computing communities in large networks using random walks. In: Yolum, p., Güngör, T., Gürgen, F., Özturan, C. (eds) Computer and Information Sciences - ISCIS 2005. ISCIS 2005. Lecture Notes in Computer Science, vol 3733. Springer, Berlin, Heidelberg.

Raghavan, U.N. *et al*. (2007) Near linear time algorithm to detect community structures in large-scale networks. *Phys Rev E Stat Nonlin Soft Matter Phys*., **76**, 036106.

Rosvall, M. and Bergstrom, C.T. (2008) Maps of random walks on complex networks reveal community structure. *Proc Natl Acad Sci U S A*., **105**, 1118-1123.

Sardina, D.S. *et al*. (2017) A novel computational method for inferring competing endogenous interactions. *Brief Bioinform*., **18**, 1071-1081.

Sarver, A.L. and Subramanian, S. (2012) Competing endogenous RNA database. *Bioinformation*, **8**, 731-3.

Shannon, P. *et al*. (2003) Cytoscape: a software environment for integrated models of biomolecular interaction networks. *Genome Res*., **13**, 2498-504.

Sumazin, P. *et al*. (2011) An extensive microRNA-mediated network of RNA-RNA interactions regulates established oncogenic pathways in glioblastoma. *Cell*, **147**, 370-81.

Tay, Y. *et al*. (2011) Coding-independent regulation of the tumor suppressor PTEN by competing endogenous mRNAs. *Cell*, **147**, 344-57.

Tucker, C.M. *et al*. (2017) A guide to phylogenetic metrics for conservation, community ecology and macroecology. *Biol Rev Camb Philos Soc*. **92**, 698-715.

Wang, N. *et al*. (2019) Single-cell microRNA-mRNA co-sequencing reveals non-genetic heterogeneity and mechanisms of microRNA regulation. *Nat Commun*., **10**, 95.

Wang, P. *et al*. (2015) miRSponge: a manually curated database for experimentally supported miRNA sponges and ceRNAs. *Database (Oxford)*, **2015**, bav098.

Wang, P. *et al*. (2022) LncACTdb 3.0: an updated database of experimentally supported ceRNA interactions and personalized networks contributing to precision medicine. *Nucleic Acids Res*., **50**, D183-D189.

Xu, J. *et al*. (2015) The mRNA related ceRNA-ceRNA landscape and significance across 20 major cancer types. *Nucleic Acids Res*., **43**, 8169-82.

Zhou, X. *et al*. (2014) Construction and investigation of breast-cancer-specific ceRNA network based on the mRNA and miRNA expression data. *IET Syst Biol*., **8**, 96-103.
